# Hydrogels Based on Poly(aspartic acid): Synthesis and Applications

**DOI:** 10.3389/fchem.2019.00755

**Published:** 2019-11-12

**Authors:** Hossein Adelnia, Idriss Blakey, Peter J. Little, Hang T. Ta

**Affiliations:** ^1^Australian Institute for Bioengineering and Nanotechnology, University of Queensland, Brisbane, QLD, Australia; ^2^School of Pharmacy, University of Queensland, Woolloongabba, QLD, Australia; ^3^Department of Pharmacy, Xinhua College of Sun Yat-sen University, Guangzhou, China

**Keywords:** hydrogels, poly(aspartic acid), poly(succinimide), crosslinking, nanoparticles, interpenetrating polymer networks (IPNs)

## Abstract

This review presents an overview on the recent progress in the synthesis, crosslinking, interpenetrating networks, and applications of poly(aspartic acid) (PASP)-based hydrogels. PASP is a synthetic acidic polypeptide that has drawn a great deal of attention in diverse applications due particularly to its biocompatibility and biodegradability. Facile modification of its precursor, poly(succinimide) (PSI), by primary amines has opened a wide window for the design of state-of-the-art hydrogels. Apart from pH-sensitivity, PASP hydrogels can be modified with suitable species in order to respond to the other desired stimuli such as temperature and reducing/oxidizing media as well. Strategies for fabrication of nanostructured PASP-based hydrogels in the form of particle and fiber are also discussed. Different cross-linking agents for PSI/PASP such as diamines, dopamine, cysteamine, and aminosilanes are also introduced. Finally, applications of PASP-based hydrogels in diverse areas particularly in biomedical are reviewed.

## Introduction

Hydrogels are 3-D networks composed of water-soluble polymer chains linked together by chemical or physical bonds. They are employed as carriers for delivery of bioactive agents, as wound healing films, bio-sensing materials, implants, and scaffolds in tissue engineering, etc. (Ullah et al., 2015; Wang L. et al., 2016; Al Harthi et al., 2019). In the presence of water, instead of dissolution, hydrogels are swollen from a few to several times of their own dry weight depending on the crosslinking degree. Similar to water-soluble polymers, based on their chemical structures, hydrogels can be divided into anionic (Ullah et al., 2015), cationic (Qi et al., 2018), non-ionic (Golabdar et al., 2019), and zwitterionic (Vatankhah-Varnosfaderani et al., 2018).

Anionic polymers, which are essentially poly(acid)s, show globule to coil transition upon pH increment and/or reduction of ionic strength (Abu-Thabit and Hamdy, 2016; Meka et al., 2017). This behavior is reflected in hydrogels as swelling when the polymer is cross-linked (Varaprasad et al., 2017). Aside from polysaccharide-based anionic polymers, most of anionic hydrogels are not biodegradable, thereby posing environmental problems and creating pollution challenges in the long-term (Guilherme et al., 2015; Pakdel and Peighambardoust, 2018). Therefore, seeking a suitable substitute that is biodegradable and non-toxic is of outmost importance.

Poly(aspartic acid) (PASP), a synthetic poly(amino acid) with a protein-like amide bond in its backbone, and a carboxylic acid as a pendant group in each repeating unit, has drawn a great deal of attention and so the demand for its production has significantly grown. The former bond provides PASP with degradability (Nakato et al., 1998; Tabata et al., 2000, 2001), while the latter groups gives the polymer acidic properties and negative charge (Yang et al., 2011; Sattari et al., 2018). Various enzymes such as trypsin (Zhang C. et al., 2017), chymotrypsin (Wei et al., 2015), dispase and collagenase I (Juriga et al., 2016), as well as different media such as activated sludge (Alford et al., 1994) and river water (Tabata et al., 2000) have been examined for biodegradation of PASP-based hydrogels and polymers. Depending on the condition (e.g., enzyme concentration and temperature), complete degradation varies from a few days to one month.

PASP hydrogels typically exhibit the same response to pH and ionic strength as other anionic ones, such that higher swelling ratio can be achieved by increasing pH or lowering ionic strength (Zhao et al., 2005; Sharma et al., 2014). This property, which is called polyelectrolyte effect, stems from ionization of carboxylic groups. Ionization (or de-protonation) creates negative charges along the chain/network, causing extended chain conformation and globule to coil transition. On the other hand, high ion concentration shields the network charge and lowers swelling (Yang et al., 2006). Additionally, by changing crosslinking density, mechanical properties and swelling could be tuned (Vatankhah-Varnoosfaderani et al., 2016). More importantly, PASP hydrogels can be readily modified with a wide variety of species to meet the need of any given application. This feature arises from highly reactive imide rings in the intermediate poly(succinimide) (PSI), which allow grafting of different molecules bearing primary amine group under mild conditions without using any catalyst. Therefore, considering facile modification, biocompatibility, and biodegradability, PASP-based hydrogels offers potential advantages over conventional anionic hydrogels [e.g., poly(acrylic acid)] and can be considered as a promising choice for hydrogel preparation in diverse applications.

In the light of the aforementioned features, in this paper we provide a review on the synthesis, gelation process, cross-linking agents, and recent applications of hydrogels based on PASP.

## Synthesis of Polyaspartic Acid

**Figure 1:**
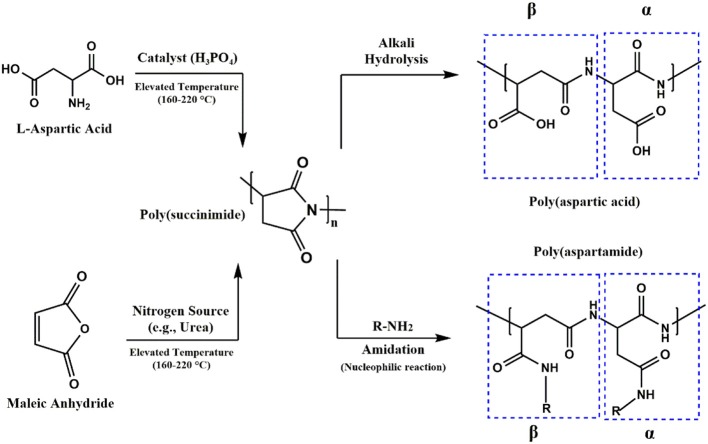
Synthesis procedure of PSI, PASP, and poly(aspartamide). PSI can either be obtained through poly-condensation (usually at elevated temperature >160°C using an acid as the catalyst) of ASP or malic acid (synthesized by maleic anhydride and a nitrogen source such as urea or ammonia). PSI can be hydrolysed under alkali media to yield PASP or reacted with primary amines (without catalyst at room temperature) to yield poly(aspartamide) derivatives. The ring opening of succinimde groups occurs both at α and β sites in amidation and hydrolysis reaction.

PASP homopolymer is generally synthesized through poly-condensation of aspartic acid (ASP) monomer or polymerization of maleamic acid which is produced from maleic anhydride and a nitrogen source like ammonia or urea as shown in Figure 1. Whatever the method is, the reaction yields the intermediate poly(anhydroaspartic acid), i.e., poly(succinimide) (PSI). The subsequent alkaline hydrolysis of PSI leads to imide ring opening through either carbonyl groups, resulting in a mixture of α and β. Also, as mentioned, PSI can easily undergo a nucleophilic reaction with primary and secondary amines without catalyst even at room temperature to yield poly(aspartamide) derivatives, allowing one to tailor-make PASP to be exploited as a versatile and multi-functional hydrogels (Feng et al., 2014; Nayunigari et al., 2014; Zhang S. et al., 2017).

### Poly-Condensation of Aspartic Acid

Thermal poly-condensation of ASP at elevated temperatures (typically higher than 160°C) can either be conducted in bulk (Nakato et al., 2000; Zrinyi et al., 2013) or in solution (Low et al., 1996; Tomida et al., 1997a) in the presence or absence of a catalyst. The reaction by-product, i.e., water, should be eliminated during the course of polymerization. The most effective solvent and catalyst have been found to be the mixture of mesitylene/sulfolane (7/3, w/w) and phosphoric acid, respectively (Tomida et al., 1997a). The reactions catalyzed by phosphoric acid yield linear chain whereas uncatalyzed reactions lead to branching (Wolk et al., 1994). High temperature, high catalyst, and aspartic acid monomer concentration can significantly increase molecular weight (Mw) (Jalalvandi and Shavandi, 2018; Yavvari et al., 2019). Recent studies have also shown when phosphoric acid is utilized as both catalyst and polymerization media (aspartic acid monomer: phosphoric acid, 1:1), PASP with high Mw and reaction yield is achieved (Zakharchenko et al., 2011; Moon et al., 2013; Szilágyi et al., 2017). It is noteworthy to mention that the use of solvent though improves heat transfer, it may reduce the reaction rate, as the availability of functional groups (NH_2_ and COOH) is reduced (Stevens, 1990). Additionally, solvent should be removed after the reaction by washing polymer. Therefore, bulk reaction under batch and continuous (through extruder) conditions is preferable in industry (Kokufuta et al., 1978; Nakato et al., 2000; Zrinyi et al., 2013).

### Polymerization of Maleamic Acid/Ammonium Salt of Maleic Acid

The second method involves polymerization of maleamic acid without catalyst for 6–8 h at high temperature (>160°C), during which period water is removed by distillation (shown in Figure 1) (Koskan and Meah, 1993; Wood, 1994; Boehmke and Schmitz, 1995; Ni et al., 2006; Shi et al., 2016). Maleamic acid is prepared by reacting maleic anhydride (MA) (or maleic acid) with anhydrous ammonia or urea as a nitrogen source or heating the monoammonium salt of maleic acid. This method was first introduced as a patent by Boehmke, where PASP was synthesized with a relatively low degree of polymerization 15–20%, using ammonia (AN) and MA which was heated in water (at 75°C) to change to maleic acid (Boehmke, 1989). The reaction is typically carried out without solvent in a reactor, oven (Freeman et al., 1995), or under microwave irradiation (Huang et al., 2007). Although this method employs industrially inexpensive and available raw materials such as maleic anhydride and ammonia, it gives low yields and low molecular weight (Boehmke, 1989; Koskan and Meah, 1993; Wood, 1994; Boehmke and Schmitz, 1995; Freeman et al., 1995; Ni et al., 2006; Huang et al., 2007; Shi et al., 2016).

## Crosslinking

Commonly, hydrogels based on PASP are prepared either via crosslinking of PSI followed by alkali hydrolysis, or by crosslinking of PASP itself. Various types of crosslinking agents can be used. Because of the simplicity, the agent is generally introduced by PSI modification or the gelation process itself is carried out on PSI followed by alkali hydrolysis.

### Hydrogels Based on Diamines

Simple succinimide ring opening by primary amines allows the use of various diamines for the synthesis of a PASP hydrogel (Jalalvandi and Shavandi, 2018). This reaction occurs at room temperature without requiring any catalyst (Fang et al., 2006a,b). Gyenes et al. (2008) employed different natural amines and amino acid derivatives such as putrescin, spermine, spermidine, lysine, and cystamine for crosslinking. They indicated that the cystamine-based hydrogels dissolve above pH 8.5 as the disulfide linkage breaks under alkaline media. Gyarmati et al. (2015) reported the synthesis of super-macroporous PASP hydrogels using 1,4-diaminobutane as a cross-linker under cryogenic condition of DMSO. Phase separation was induced by freezing DMSO as the solvent of PSI. As a result, highly porous interconnective hydrogels (pore size 9–259 μm) was fabricated, which is useful for *in vitro* cell seeding with pH-induced detachment of the grown cells. In a similar study, aside from chemical crosslinking with hexamethylenediamine (HMDA), freeze/thaw technique was also applied to induce phase separation and physical crosslinking (Zhao and Tan, 2006). Swelling behavior was highly affected by changing freeze/thaw cycle number, time, and temperature. Chen et al. (2016) also prepared PASP superabsorbent cross-linked by HMDA in the presence of organic bentonite (OB) with high swelling capacity (491 g/g in water). It was shown that OB can serve as a crosslinker due to its surface amine groups since high OB content (above 3%) led to lower swelling.

### Hydrogels Based on Disulfide Bond

**Figure 2:**
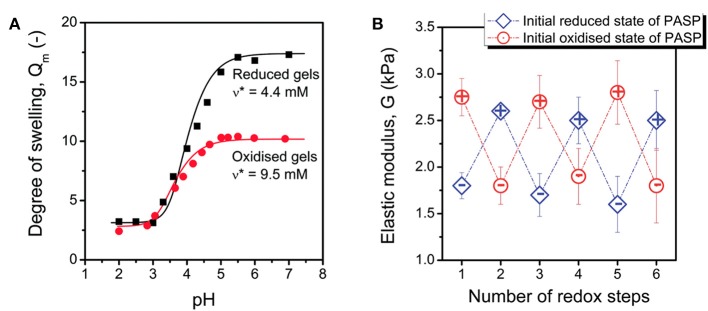
PASP hydrogels based on disulfide bonds. **(A)** Swelling of PASP hydrogels cross-linked with cysteamine in reduced and oxidized states as a function of pH shows conventional behavior of anionic hydrogels, swelling under reduced condition is higher. **(B)** Elastic modulus of the corresponding hydrogels shows reversible increase and decrease upon oxidation/reduction. Reproduced from Gyarmati et al. (2014) with permission from The Royal Society of Chemistry.

**Figure 3:**
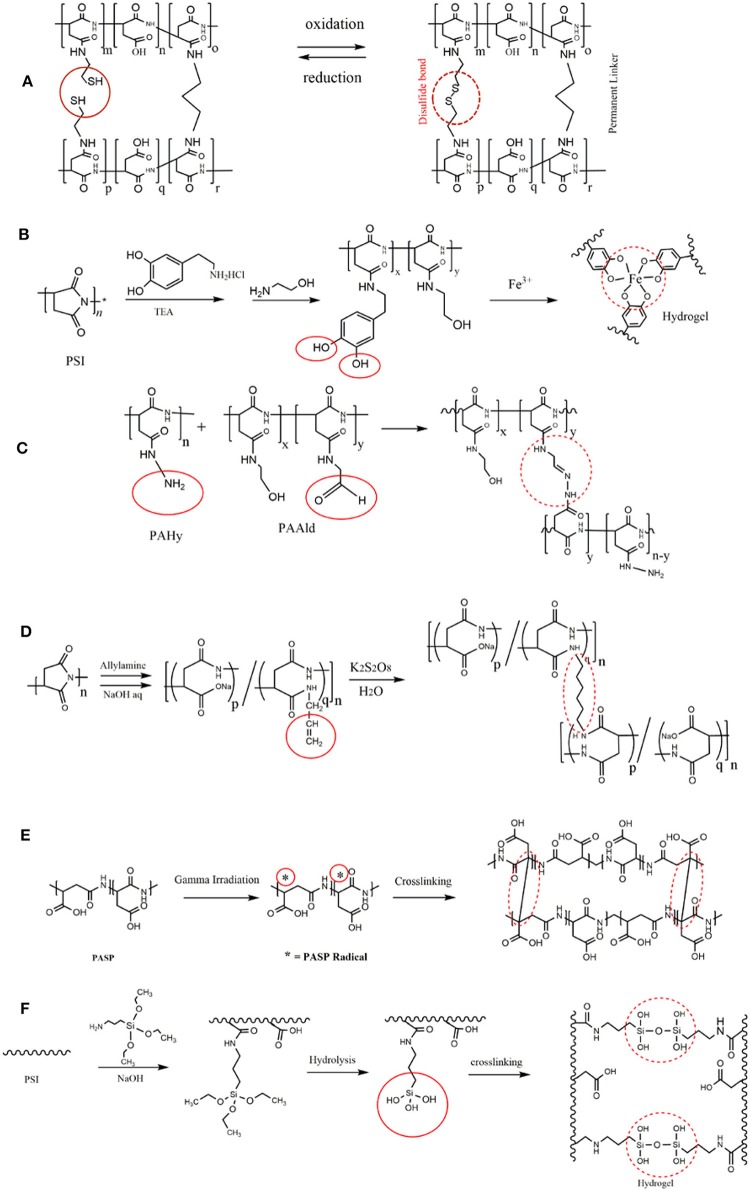
PASP hydrogels prepared by different cross-linkers. **(A)** PASP hydrogel with a permanent and a cleavable cysteamine cross-linker exhibited response to oxidation/reduction without the gel dissolution. **(B)** Modification of PSI with dopamine led to PASP with catechol pendant groups which can establish complex with Fe^3+^ ions, and form hydrogel as a results; adapted from Gong et al. (2017) with permission from Wiley. **(C)** Synthesis of injectable PASP hydrogels by introduction of aldehyde and amine groups on PASP. Reproduced from Lu et al. (2014) with permission from Wiley. **(D)** Allyl amine as a monomer that is polymerized via radical polymerization is grafted onto PASP; the subsequent polymerization of allyl amine gives rise to PASP-based hydrogel. **(E)** Application of gamma-irradiation for crosslinking of PASP. **(F)** Grafting of APTS as an aminosilane on PSI backbone followed by its hydrolysis.

Crosslinking through disulfide or thiol containing agents endows an interesting feature to the PASP-based hydrogels. The reaction of thiol to disulfide can be carried out under application of a reducing agent. This reaction can be reversed in the presence of an oxidizing agent. Therefore, PSI is generally modified with thiol groups (cysteamine or cystamine) for the preparation of reducing/oxidizing-responsive PASP hydrogels (Molnar et al., 2014). In order to maintain structural integrity in different media, a permanent linker such as a diamine can be employed (**Figure 3A**; Zrinyi et al., 2013; Krisch et al., 2018). Recently, such dual cross-linked hydrogels have drawn a great deal of attention due to swelling under reductive state. For instance, Zrinyi et al. (2013) synthesized PASP with diaminobutane (DAB), and cystamine (CYS) as permanent and cleavable crosslinkers, respectively. They showed that disulfide bonds arising from the latter is broken by the addition of a reducing agent, leading to an increase in swelling and a decrease in modulus. Likewise, redox- and pH-responsive PASP hydrogels were prepared by dual crosslinking using cysteamine, and 1,4-diaminobutane which creates reversible and irreversible bonds, respectively (Gyarmati et al., 2014). It was indicated that swelling degree of hydrogel and elastic modulus can be tuned by reducing/oxidizing agents without hydrogel disintegration/dissolution. Swelling increased as pH increased both under oxidized and reduced states. However, under the latter condition, swelling was higher. The hydrogels maintained their mechanical stability under repeated redox cycles for at least three cycles and the reversibility was shown to be independent of initial redox state of PASP (reduced or oxidized) (Figures 2A,B). Krisch et al. (2018) employed poly(ethylene glycol) diglycidyl ether (PEGDGE) for crosslinking thiolated PASP in order to secure structural integrity of the hydrogels in reducing media. A part of thiol groups were reacted with the former to establish a non-cleavable gel junction while the remaining ones were oxidized into breakable disulfide bonds. It should be noted that the epoxide groups with thiol groups form unbreakable S-C bonds.

### Hydrogels Based on Dopamine

Catechol moieties in dopamine exhibit a multifunctional characteristic for the design of mussel-inspired coatings (Ryu et al., 2015, 2018; Saiz-Poseu et al., 2019). Complex formation of catechol with boron and/or iron ions (Fe^3+^) can be employed for hydrogel preparation (Vatankhah-Varnoosfaderani et al., 2014; Krogsgaard et al., 2016). Injectable dopamine modified PASP hydrogels with superior adhesive character were synthesized by complexation with Fe^3+^ ions (gelation time around 1 min; Figure 3B; Gong et al., 2017). It was suggested that the resulting crosslinking are composed of both Fe3^+^ coordination as well as covalent quinone-quinone bonds. Boric acid was also shown to crosslink dopamine-modified PASP and yield hydrogels due to boron–catechol coordination (Wang B. et al., 2016). The prepared hydrogels had autonomous self-healing feature due to such a coordination.

### Other Hydrogels

Apart from diamine and disulfide crosslinking, other strategies have also been developed for fabrication of PASP hydrogels. The reaction of hydrazine and aldehyde, radical polymerization of pendant double bond, sol-gel reaction of aminosilane, and application of gamma-irradiation are some examples that will be discussed in this section for preparation of PASP hydrogels.

Lu et al. (2014) prepared injectable PASP-based hydrogel through introduction of hydrazine and aldehyde to PSI backbone by hydrazine hydrate and 3-amino-1,2-propanediol, respectively. Therefore, hydrazine and aldehyde modified PASPs were used as two gel precursors as shown in Figure 3C. The same strategy was used for crosslinking of oxidized alginate and polyaspartamide conjugated with RGD peptide (Jang and Cha, 2018).

Conventional radical polymerization of allyl amine monomer grafted onto PSI can also lead to crosslinking (Figure 3D; Umeda et al., 2011; Némethy et al., 2013). Minimum value of allyl amine content was found to be 5% for gel formation (Umeda et al., 2011).

Gamma-irradiation can be typically utilized for crosslinking of polymers as it delivers high amount of energy and is capable of forming free radical on polymer backbone. Using such radiation (dosage of 32–100 kGy), Tomida et al. (1997b) prepared PASP hydrogel (Figure 3E). It was shown that the reaction should be conducted under N_2_ atmosphere as oxygen scavenges free radicals. It was also found that low polymer concentration, as well as low Mw does not lead to gelation and also acidic conditions destabilize the generated radicals.

γ-aminopropyltriethoxysilane (APTS) (an aminosilane) is generally used for attachment of organic/inorganic materials, and surface modification (Adelnia et al., 2015; Bidsorkhi et al., 2017). Its amine and hydroxyl groups make it an excellent candidate as a linker. Meng et al. (2016) introduced APTS on PSI backbone and used it as a crosslinker for PASP gel formation (Figure 3F).

Ethylene glycol diglycidyl ether (EGDGE) can also react with PASP to yield hydrogels (at 180°C for 30 min, dry state, pH before drying 5–6.5) (Chang and Swift, 1999). As the degree of ionization of PASP as well as the protonation of epoxide ring is highly dependent on pH, crosslinking occurs at optimum pH of 5–6.5. Acidic media hydrolyse epoxide group whereas alkaline media reduce the protonated acid group concentration required for nucleophilic attack on the epoxide ring. Meng et al. (2015) however, utilized EGDGE for PSI crosslinking and compared it with hydrazine as a diamine. The produced bonds of the former and the latter are ester and amide, respectively. The PASP hydrogels with the latter had faster swelling kinetic, while lower stability in terms of maintaining the absorbed water.

## PASP Hydrogel Nanostructures

### PASP Hydrogel Particles (i.e., Nanogels and Microgels)

**Figure 4:**
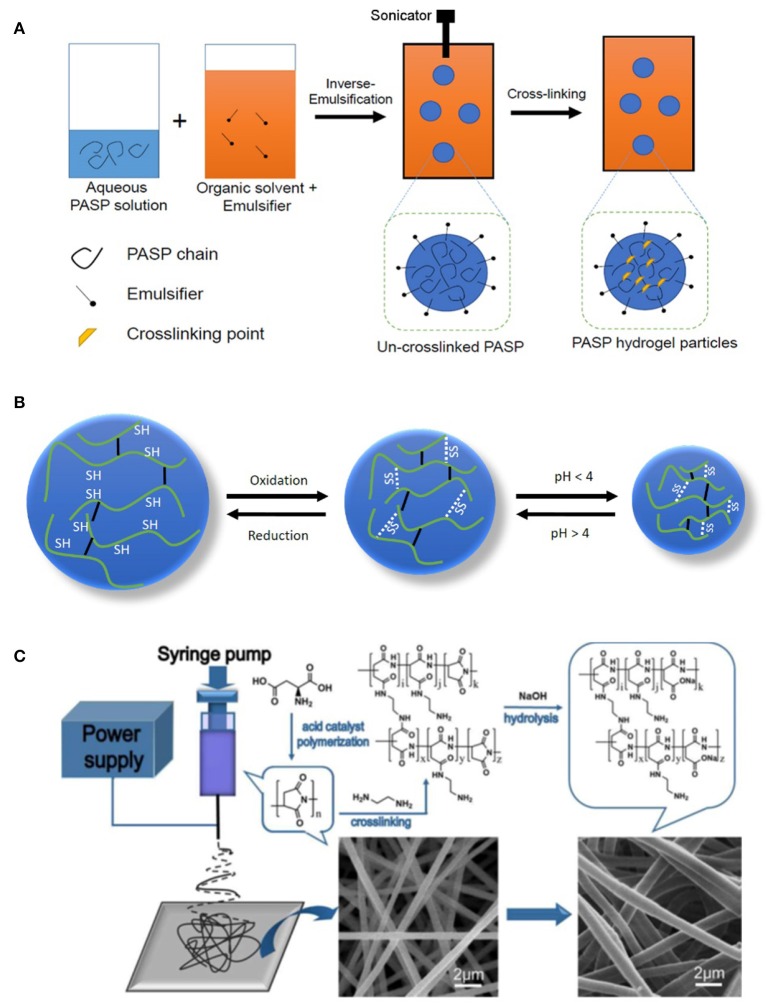
Preparation of nanostructured PASP hydrogels. **(A)** Schematic representation of the preparation of PASP-based nanogels in inverse emulsion technique. **(B)** Schematic representing volume change of pH- and redox/reductive-responsive nanogels, white dashed lines represent cleavable disulfide bonds while solid black lines stand for permanent linker; oxidation leads to shrinkage of nanogels as crosslinking density increases, while acidic pH values protonate COOH groups of PASP, lowering the swelling. **(C)** Schematic representation of the method for PASP fiber hydrogel formation. Reproduced from Zhang et al. (2015) with permission from Elsevier.

Hydrogel nanoparticles (i.e., nanogels) can find much more applications compared to their own bulk counterpart especially as a carrier for delivery of bioactive agents (Cuggino et al., 2019; Molaei et al., 2019). Preparation of such structures is generally carried out via inverse type emulsion techniques where aqueous phase containing hydrophilic polymer is dispersed in an organic solvent (typically hydrocarbons such as hexane) containing emulsifier (e.g., span-80; Krisch et al., 2017). Formation of small droplets/particles requires high shear stress which can be applied through high speed homogenizer or sonication. Schematic representation of typical emulsification is drawn in Figure 4A. Network formation should be conducted after particle formation as premature crosslinking leads to bulk gelation and does not allow emulsification. For example, Krisch et al. (2016) prepared nanogels of thiolated PASP by inverse miniemulsion (water in n-hexane). After particle formation, the thiolated groups of PASP were oxidized by sodium bromate NaBrO_3_, giving rise to gel formation. To maintain structural integrity of nanogels in reducing media, the same group utilized poly(ethylene glycol) diglycidyl ether for crosslinking of a part of S-H groups. The nanogels diameter increased in reducing media due to disulfide bond breakage and thus swelling while maintaining nanogel integrity (Figure 4B; Krisch et al., 2018).

Inverse emulsion method, though effective in preparation of small size and uniform particles, is accompanied with several problems such as using toxic organic solvent, relatively high amount of emulsifier, and the need for medium substitution to water. Therefore, alternative methods need to be adopted. Since PSI is hydrophobic, its particles can be formed in aqueous media (Hill et al., 2015). PASP-g-PEG hydrogel nanoparticles were fabricated via self-association of hydrophobic PSI units in water (i.e., micelle formation), followed by their hydrolysis (Park et al., 2017). The particles were crosslinked with HMDA or cystamine. PASP particles can also be formed via self-assembly with cationic polymers such as chitosan, as it is an anionic polyelectrolyte. Such an electrostatic self-assembly (also referred to as ionic gelatification) can yield composite nanoparticles in water through polyelectrolyte complexation (i.e., electrostatic charge attraction of the two polymer; Zheng et al., 2007; Zhang et al., 2008; Wei Wang et al., 2009). PASP/chitosan particles were prepared by drop-wise addition of chitosan to PASP solution. When chitosan/PASP ratio increased from 0.75 to 2.5, the size increased from 84 to 1,364 nm (Hong et al., 2014).

### PASP Hydrogel Nanofibers

Regarding the fiber formation, since gels cannot flow, network formation should be conducted after fiber formation similar to the emulsification mentioned above. A typical PASP-based fiber preparation is exhibited in Figure 4C. In this method, PSI is dissolved in its solvent (commonly DMF or DMSO) and electrospun into nanofibers. The resulting PSI nanofibers are cross-linked in this step, employing a suitable agent (e.g., ethylenediamine) and converted to PASP by alkali hydrolysis (Zhang et al., 2015; Zhang C. et al., 2017). Interestingly, Zhang C. et al. (2017) found that inter-fiber crosslinking can also occur, resulting in hydrogels with interconnected microporous structure. This causes higher deformation, swelling kinetic, and swelling ratio compared to hydrogel films. Another study revealed that crosslinking can also be carried out during electrospinning for cysteamine-grafted PASP (diameter 80–500 nm; Molnar et al., 2014). However, relatively lower polymer concentration (15 wt.%) compared to conventional electrospinning process should be used to avoid premature gelation.

## Interpenetrating Polymer Network (IPN) Based on PASP

Interpenetrating polymer networks (IPN) are a class of materials composed of two chemically distinct, but highly compatible polymers that are uniformly mixed in each other in microscopic scales without any phase separation. IPNs are divided into semi-IPNs and full IPNs, in which one or both components are cross-linked, respectively (Roland, 2015). IPNs are generally fabricated to take advantage of the features of both components.

**Figure 5:**
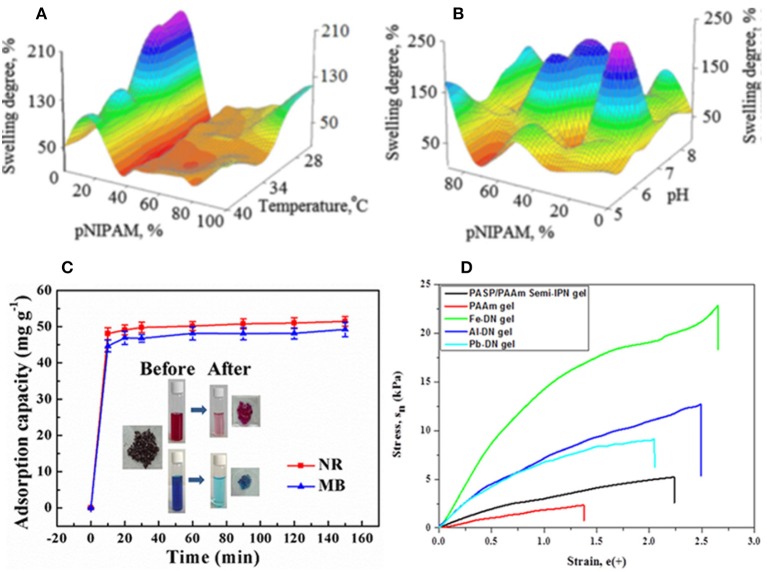
Properties of IPN hydrogels based on PASP. **(A,B)** 3D swelling degree dependence on the network components and test parameters: **(A)** Temperature and **(B)** pH. Reproduced from Nistor et al. (2013) with permission from Wiley. **(C)** Adsorption capacity of methylene blue (MB) and neutral red (NR) by PASP/PAA semi-IPN hydrogels. Reproduced from Jv et al. (2019) with permission from American Chemical Society. **(D)** Stress-strain curve of double networks of PASP/PAAm hydrogels in the presence and absence of metallic cations. Reproduced from Lee et al. (2018) with permission from Wiley.

For instance, poly(N-isopropylacrylamide) [poly(NIPAAm)] which is a well-known polymer with LCST at around physiological temperature, can be introduced for providing the IPN with temperature sensitivity. Liu et al. (2012) prepared NIPAAm/PASP IPN hydrogels that show response both to pH and temperature. They first cross-linked PSI with a diamine, followed by its hydrolysis to PASP hydrogel. The hydrogel was then swelled with NIPAAm monomer/N,N′-methylene bisacrylamide (MBA) crosslinker followed by their polymerization. Némethy et al. (2013) synthesized NIPAAm/PASP co-network hydrogels by grafting allyl amine monomer onto PSI backbone followed by its radical polymerization with NIPAAm, and PSI hydrolysis. Nistor et al. (2013) evaluated swelling degree of PASP/PNIPAAm semi-IPN as a function of pH, temperature and NIPAAm content (Figures 5A,B). Other polymers including poly(vinyl alcohol), poly(acrylic acid), and poly(acrylamide) have also been employed for PASP-based IPN preparation as summarized in Table 1. Zhao et al. (2006) introduced PAA to PASP-based semi-IPN hydrogels by polymerization of acrylic acid and MBA as a cross-linker in the PASP solution. It was found that the swelling ratio increases with increasing PASP content as well as temperature (range of 40–60°C). The incorporation of high Mw PASP also inhibited gel formation due to steric hindrance. Jv et al. (2019) indicated that semi-IPNs based on PASP/PAA possess excellent ability for removal of methylene blue and neutral red with maximum adsorption of 357.14 and 370.37 mg/g, respectively (Figure 5C). Magnetic nanoparticles of Fe_3_O_4_, were incorporated into the hydrogels for facile separation of the dye-containing solid. Lee et al. (2018) exhibited that PASP improves mechanical properties of brittle PAAm significantly. They suggested that the addition of multivalent cations such as Fe^3+^, Al^3+^, Pb^2+^, Cu^2+^ results in ionic coordination, and thus creation of second network. Iron cation (Fe^3+^) had the highest impact on improving mechanical properties (Figure 5D).

**Table 1:** PASP-based IPN and semi-IPN with different polymers (denoted as polymer 2).

	**Polymer 2**	**X-linker1**	**X-linker2**	**Type**	**Characteristic**	**References**
1	N-isopropylacrylamide	1,4-diaminobutane (DAB)	N,N′-methylene bisacrylamide (MBA)	IPN	Excellent pH-responsiveness	Némethy et al., 2013
		Hexamethylenediamine (HMDA)	MBA	IPN	Dual pH- and temperature- sensitive hydrogel. Large porous structure; fast shrinking and re-swelling	Liu et al., 2012
			Diethylene Glycol diacrylate (DEGDA)	Semi-IPN	Dual pH- and temperature-sensitive hydrogel	Nistor et al., 2013
2	Acrylic acid		MBA	Semi-IPN	The freezing temperature resulted in a more porous hydrogel and faster swelling/deswelling rates	Lim et al., 2016
			MBA	Semi-IPN	Improving responsive behavior of the hydrogel to alternating changes in inorganic salt, pH, and temperature	Zhao et al., 2006
			MBA	Semi-IPN	The hydrogels had dye pollutant removal ability. Magnetic NPs were added for separation of the solid after dye removal	Jv et al., 2019
			MBA	Semi-IPN	Polygorskite clay caused higher swelling rate than that of pure hydrogels of PAA/PASP	Ma et al., 2015
			MBA and Ethylene glycol dimethylacrylate (EGDMA)	Semi-IPN	MBA and EGDMA resulted in higher swelling behavior in acidic and basic medium, respectively	Sharma et al., 2016
3	Acrylamide		MBA	Semi-IPN	Metal cations (Fe^3+^, Al^3+^, Pb^2+^, Cu^2+^) led to creation of second network, and increased mechanical strength and decreased swelling ratio of the gel	Lee et al., 2018
4	poly(vinyl alcohol)	γ-aminopropyltriethoxysilane (APTS)		Semi-IPN and IPN	Interpenetrating PASP/PVA hydrogel resulted in higher, faster swelling ratio, and higher drug releasing	Lu et al., 2015

## PASP Hydrogel Applications

Apart from conventional and common applications of hydrogels such as hygiene, and agricultural products, PASP hydrogels can be utilized in a wide variety of biomedical engineering areas such as development of scaffolds for tissue engineering, and carriers for sustained or targeted drug delivery systems (DDS). This is mainly due to its biocompatibility, biodegradability, as well as stimuli-responsive characteristic. Regarding the latter, in particular pH- and redox/oxidation-sensitivity of PASP has been exploited for DDS (Horvát et al., 2015; Sim et al., 2018). In this section, some recent studies in these regards are presented.

**Figure 6:**
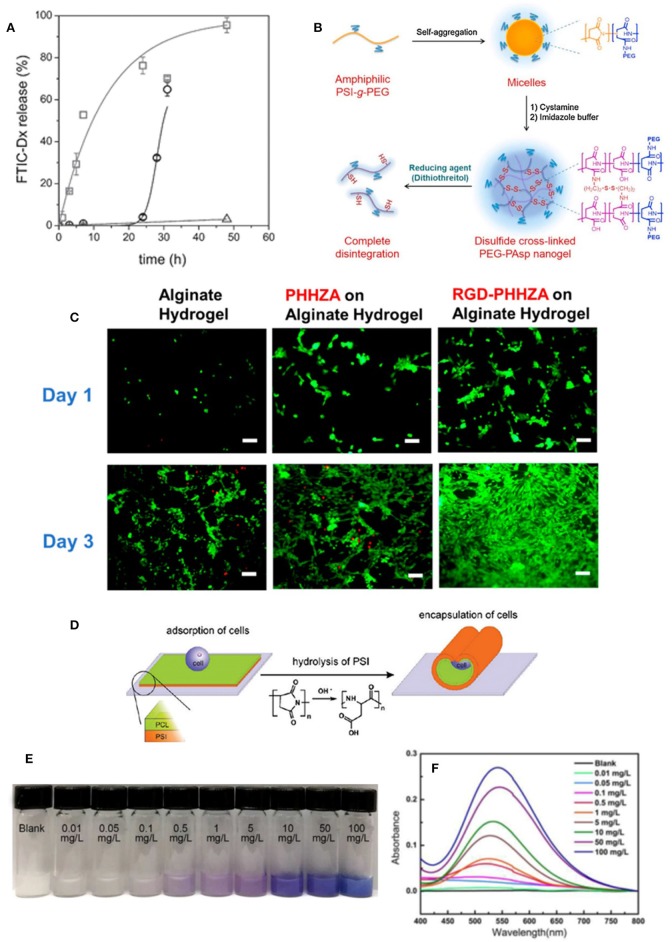
Biomedical and industrial applications of PASP-based hydrogels. **(A)** FITC-Dx release from PASP nanogels as a function of time and DTT concentration (squares: 100 × 10^−3^ M, spheres: 10 × 10^−3^ M, and triangles: 0 × 10^−3^ M DTT). Reproduced from Krisch et al. (2016) with permission from Wiley. **(B)** Synthesis of the disulfide cross-linked PASP nanogel and its complete disintegration by reducing agent. Reproduced from Park et al. (2017) from Elsevier. **(C)** The live (green) and dead (red) cells cultured on alginate, alginate/poly(2-hydroxyethyl-co-hydrazidoadipoyl aspartamide) (PHHZA), and alginate/RGD-PHHZA hydrogels after 1 and 3 days (scale bar: 100 μm). Reproduced from Jang and Cha (2018) with permission from American Chemical Society. **(D)** The schematic illustration of cell encapsulation by bilayer PSI/PCL tubes. Reproduced from Zakharchenko et al. (2011) with permission from American Chemical Society. **(E)** Color change and **(F)** absorption spectra of PASP in different Cu^2+^ ions solution. Reproduced from Zhang et al. (2015) from with permission Elsevier.

PASP/PNIPAAm co-network hydrogels loaded with sodium diclofenac (DFS) showed pH sensitivity such that the release of DFS increased when the gel is delivered from stomach (pH 1.2) into the bowels (pH 7.6) (Némethy et al., 2013). Such a conventional pH sensitivity feature can protect both the stomach from the side effects of DFS and the drug itself from acidity of stomach. However, in another study, unusual pH-response was observed in PASP hydrogels cross-linked with hydrazine and aldehyde (Lu et al., 2014). The release rate of DOX was accelerated by decreasing pH from 7 to a weak acidic condition (ca. pH 5). This behavior was attributed to instability of the hydrazone bond in acidic media, resulting in loosening of gel network. DFS was also employed as an ocular drugs and loaded in *in-situ* gelling thiolated PASP for its sustainable delivery (Horvát et al., 2015). The polymer due to its negative charge showed strong mucoadhesion, as well as high resistance against lachrymation of the eye. This is attributed to mucin glycoproteins role for crosslinking (i.e., disulfide linkage). The drug release showed a burst-like profile in the first hour followed by sustained release up to 24 h. In another work, fluorescent dextran (FTIC-Dx) was loaded into thiolated PASP nanogels prepared by inverse emulsion (Krisch et al., 2016). Disulfide bonds were cleaved by a reducing agent for gel disintegration, and release of the loaded drug. As seen in Figure 6A, the release profile dramatically increased by the addition of DTT as a reducing agent. The same redox-response and DOX release was seen in thiolated PASP-g-PEG nanogels (Park et al., 2017). Under reductive intracellular conditions, the prepared nanogels were shown to have the ability to release DOX and efficiently translocated to the nucleus of cancer cells (Figure 6B). Epigallocatechin Gallate (EGCG) which is the main bioactive element of green tea and is unstable *in vitro* was encapsulated in PASP/chitosan particles (Hong et al., 2014). The release of EGCG was investigated by simulation of food ingestion pH condition. It was demonstrated that EGCG is much more effective against rabbit atherosclerosis when encapsulated into PASP/chitosan.

Jang and Cha (2018) incorporated RGD peptide to PSI for improving 3T3 fibroblast cell adhesion. PSI was further modified with hydrazide, and subsequently reacted with oxidized alginate, bearing aldehyde groups. The Schiff reaction (i.e., aldehyde-hydrazide, yielding hydrazone bond) leads to *in-situ* gelation of poly(aspartamide)/alginate. As shown in Figure 6C, RGD-modified hydrogels possessed much better cell viability, adhesion and proliferation compared to un-modified hydrogels. Juriga et al. (2016) also modified thiolated PASP hydrogels with RGD and utilized them as scaffolds for MG-63 osteoblast-like cells. It was shown that RGD introduction leads to compacted cluster formation of the cells. The prepared scaffolds provided the osteoblast-like cells with excellent condition for adhesion, viability, and proliferation.

Zakharchenko et al. (2011) fabricated polymer tubes and encapsulated yeast cells within them. The tubes composed of bilayer cross-linked films of PSI/polycaprolactone. Upon the hydrolysis of PSI in physiological buffer environment, and conversion into PASP gels, the films self-rolled due to the produced internal stress as a result of swelling of the lower layer, i.e., PASP (Figure 6D). Such a self-rolling was exploited for cell encapsulation. Hydrolysis of PSI was shown to be step like process and initiates after nearly 8 h in PBS buffer.

PASP due to its negative charge can endow electrostatic stability to colloidal systems. For example, iron oxide (Fe_3_O_4_) nanoparticles coated with a thin layer of PASP hydrogels had improved colloidal stability (Vega-Chacón et al., 2017). The composite magnetic particles did not show any adverse effect on cell viability of L929 fibroblast. Also, particles exhibited response to pH, presenting them as promising candidate for magnetic drug delivery. Iron oxide nanoparticles as negative contrast agents for magnetic resonance imaging (MRI) have been employed widely for detection of diseases (Ta et al., 2011, 2017a,b, 2018; Gaston et al., 2018; Wu et al., 2018; Yusof et al., 2019; Zhang et al., 2019). Multifunctional PASP nanoparticles containing iron oxide nanocrystals and doxorubicin was also developed for simultaneous diagnosis and treatment of cancer by Yang et al. (2011). Iron oxide nanocrystals were loaded in PASP nanoparticles through an emulsion method using octadecyl grafted PASP, then doxorubicin (DOX), was incorporated in the magnetic PASP nanoparticles. It was shown that the DOX loaded nanoparticles exhibited high *T*_2_ relaxivity and strong cytotoxicity for cancer cells.

Due to its strong ability for chelation, PAPS nanofiber hydrogels were utilized as chemosensor for Cu^2+^ ions detection (Zhang et al., 2015). The hydrogels showed high sensitivity and selectivity to Cu^2+^ ions compared with other ions such as Ag^+^, and Ca^2+^ where no color change was observed (Figures 6E,F). The detection limit of as low as 0.01 mg/L was reported.

Because of its bio-degradation and water uptake, PASP hydrogels could be regarded as a promising candidate for ecological restoration and plant survival especially in arid area. Wei et al. (2016) employed PASP hydrogel to transplant Xanthoceras sorbifolia seedlings. The survival rate and the leaf water content were improved in soils containing PASP hydrogels.

## Conclusion and Prospects

Although synthesis of PASP-based hydrogels is relatively more complex than other anionic-based hydrogels such as PAA-based ones, its biocompatibility and biodegradability make it attractive particularly in biomedical applications. Though pH-responsive, PASP is further modified with other moieties to provide sensitivity to the desired stimuli as well including temperature and reducing/oxidizing media. Incorporation of other water-soluble polymers into the PASP network may also provide the final hydrogel with superior properties. Scrutinizing the literature, it is found that PASP hydrogels has not yet been employed for inhibition of scale formation in which PASP solution has exhibited promising results (Hasson et al., 2011). Moreover, PASP hydrogel fibers may potentially be a good candidate as scaffold for cell culture as well as tissue engineering. Additionally, due to its anionic nature, PASP-based hydrogels can be used for preparation of electrically-responsive materials (Murdan, 2003).

## Author Contributions

HA wrote and revised the manuscript. IB and PL supervised the work. HT supervised and revised the manuscript.

### Conflict of Interest

The authors declare that the research was conducted in the absence of any commercial or financial relationships that could be construed as a potential conflict of interest.

## Abbreviations

APTS: γ-aminopropyltriethoxysilane
AN: ammonia
ASP: Aspartic acid
CYS: cystamine
DAB: 1,4-diaminobutane
DEGDA: diethylene glycol diacrylate
DTT: dithiothreitol
DDS: drug delivery system
EGDGE: ethylene glycol diglycidyl ether
EGDMA: ethylene glycol dimethylacrylate
FTIC-Dx: fluorescent dextran
HMDA: hexamethylenediamine
MA: maleic anhydride
MBA: N,N′-methylene bisacrylamide
MB: methylene blue
NR: neutral red
PAAm: poly(acrylamide)
PASP: poly(aspartic acid)
PEGDGE: poly(ethylene glycol) diglycidyl ether
poly(NIPAAm): poly(N-isopropylacrylamide)
PSI: poly(succinimde)
TMEDA: tetramethylenediamine (TMEDA).
